# Dual PI3K and Wnt pathway inhibition is a synergistic combination against triple negative breast cancer

**DOI:** 10.1038/s41523-017-0016-8

**Published:** 2017-04-26

**Authors:** Jeffrey P. Solzak, Rutuja V. Atale, Bradley A. Hancock, Anthony L. Sinn, Karen E. Pollok, David R. Jones, Milan Radovich

**Affiliations:** 1 Department of Surgery, Indiana University School of Medicine, Indiana University Melvin and Bren Simon Cancer Center, Indianapolis, IN USA; 2 In Vivo Therapeutics Core, Indiana University Melvin and Bren Simon Cancer Center, Indiana University School of Medicine, Indianapolis, IN USA; 3 Herman B. Wells Center for Pediatric Research, Department of Pediatrics, Section of Pediatric Hematology/Oncology, Riley Hospital for Children at Indiana University Health Department of Pharmacology and Toxicology, Indianapolis, IN USA; 4 Department of Medicine, Division of Clinical Pharmacology, Indiana University School of Medicine, Indianapolis, IN USA

## Abstract

Triple negative breast cancer accounts for 15–20% of all breast cancer cases, but despite its lower incidence, contributes to a disproportionately higher rate of mortality. As there are currently no Food and Drug Administration-approved targeted agents for triple negative breast cancer, we embarked on a genomic-guided effort to identify novel targeted modalities. Analyses by our group and The Cancer Genome Atlas have identified activation of the PI3K-pathway in the majority of triple negative breast cancers. As single agent therapy is commonly subject to resistance, we investigated the use of combination therapy against compensatory pathways. Herein, we demonstrate that pan-PI3K inhibition in triple negative breast cancers results in marked activation of the Wnt-pathway. Using the combination of two inhibitors currently in clinical trial as single agents, buparlisib(pan-PI3K) and WNT974(WNT-pathway), we demonstrate significant in vitro and in vivo synergy against triple negative breast cancer cell lines and xenografts. Taken together, these observations provide a strong rationale for testing dual targeting of the PI3K and WNT-pathways in clinical trials.

## Therapeutics: Dual therapy shrinks triple negative breast tumors

Two experimental drugs currently in clinical trials as single agents might work better together against triple negative breast cancer. Jeffrey Solzak and Milan Radovich, and colleagues from the Indiana University School of Medicine in Indianapolis, USA, analyzed gene expression data from cancerous and healthy breast tissues and from published genomic datasets. They found the PI3K pathway consistently activated in the majority of breast cancers that test negative for the three most common receptors known to fuel breast tumor growth. Inhibiting PI3K in these cells led to a marked increase in the activity of another critical pathway called Wnt, an observation that prompted the researchers to test a combination of a PI3K inhibitor, buparlisib, and a Wnt inhibitor, WNT974, in cell lines and xenograft models. Tumor cells shrunk more and mice lived longer than treatment with either agent alone.

## Introduction

Triple negative breast cancer (TNBC) accounts for 15% of all breast cancer cases in the United States, and despite its lower incidence, contributes to a disproportionately higher rate of mortality compared to other breast cancer subtypes. Because these tumors lack over-expression of the estrogen, progesterone, and HER-2 receptors (“triple-negative”), these patients do not respond to targeted therapies that have been successfully used against tumors that over-express these proteins.^1–5^ Agents such as tamoxifen, the aromatase inhibitors, and trastuzumab (Herceptin) have made major advances in improving survival for hormone-positive and HER-2 positive breast cancers, but successful targeted therapies have been absent in TNBC. Treatment options are very limited to TNBC patients beyond standard chemotherapy.^6^ Particularly in the curative setting, the drugs that comprise adjuvant and neoadjuvant chemotherapy for TNBC (anthracyclines, cyclophosphamide, taxanes, and platinum) have existed for decades. Previous attempts to introduce targeted therapies in TNBC by inhibiting the EGFR and c-KIT receptors have resulted in negative clinical trials.^7–11^ More recent attempts to introduce PARP inhibitors for TNBC have demonstrated some clinical success, though it may be limited to specific BRCA1 subpopulations.^12–14^ Very recent data targeting the androgen receptor (AR) with enzalutamide in a small subtype of TNBCs that are AR-positive showed some promising clinical results, but the efficacy is restricted to a limited subpopulation.^15^ Recent excitement with immune therapy has spurred on efforts to test immune checkpoint therapy in TNBC. The phase Ib JAVELIN trial using avelumab, an anti-PD-L1 antibody, showed a low overall response rate of 8.6% in patients with metastatic TNBCs, but the responses were durable.^16^ In another phase Ib trial using pembrolizumab in advanced TNBC, results demonstrated an 18.5% overall response rate, with a subset also having a long durable response.^17^ While targeted therapies are available on trial for niche populations of TNBCs, a critical need exists to identify novel therapies for the vast majority of patients with this disease. In an effort to identify novel targets for TNBC, our group has previously published a comprehensive transcriptional compendium of 94 TNBCs compared to microdissected normal breast tissue using next-generation RNA-sequencing.^18^ Subsequent analyses of this data using novel network analysis tools, has identified activation and over-expression of the PI3K/AKT/mTOR (PI3K) pathway and the Wnt Pathway. The PI3K pathway is well known for its role in cellular proliferation and survival,^19^ and the Wnt pathway is well known for its role in tissue development.^20^ Early stage inhibitors of these two pathways are currently in clinical trials (clinicaltrials.gov). Buparlisib (a pan-PI3K inhibitor), and WNT974 (a Wnt pathway inhibitor) were obtained to perform in vitro and in vivo testing. Herein, we will show that treatment with the combination of buparlisib and WNT974 is highly synergistic in reducing TNBC viability. In addition, our data demonstrates that treatment with buparlisib, induces compensatory expression of Wnt pathway proteins namely, porcupine (the protein target for WNT974), whose O-acyltransferase activity is required for Wnt ligand pamitoylation and secretion.^21^ These data demonstrate a potential mode of synergy for these two agents and may provide a viable therapeutic option for patients with TNBC.

## Results

### RNA sequencing identifies overexpressed PI3K pathway

**Fig. 1:**
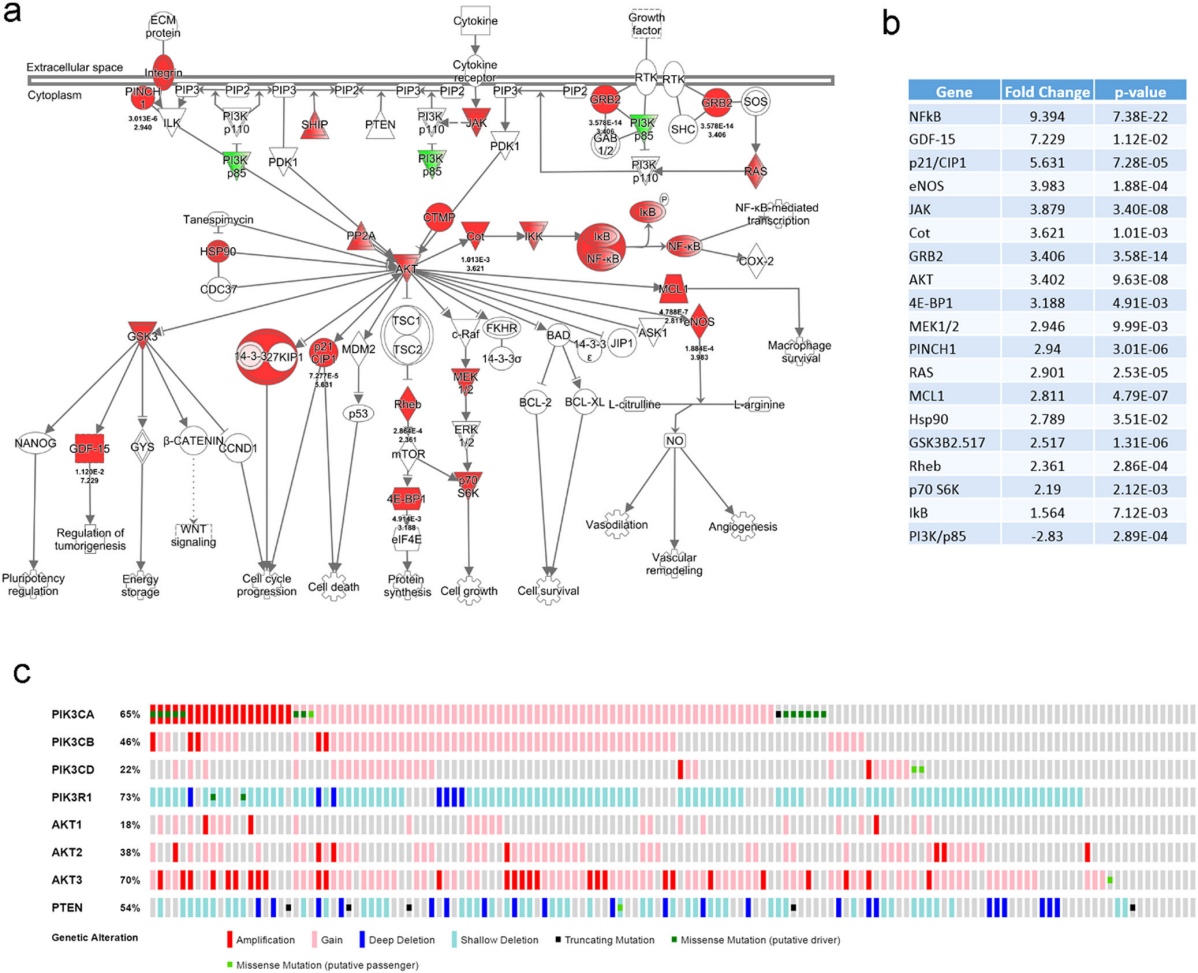
**a** Pathway analysis of RNA-seq data comparing 94 TNBCs vs. 20 normal breast tissues reveals the overexpression of the PI3K/AKT pathway. Statistical analysis revealed 3,197 genes (FDR < 5%, with a fold change more than ±2) that are differentially expressed between TNBC and normal (Supplementary Table 1). Significant genes were imported into ingenuity pathway analysis, revealing an active PI3K/AKT pathway. **b** The gene expression fold-changes and *p*-values of the differentially expressed components of the PI3K pathway in 1 **a**. **c** TCGA analysis using the cBioPortal database of 139 TNBC patients show that approximately 92% of individuals have an observed genomic aberration within the PI3K/AKT pathway. In this oncoprint plot, each row represents the stated gene, and each column is an individual patient. Colors representing each form of aberration are depicted below the plot

RNA sequencing data comparing 94 TNBCs to 20 normal breast tissues (microdissected ductal epithelium from healthy volunteers) were analyzed to identify differentially expressed genes, as previously described.^18^ When considering a false discovery rate (FDR) < 0.05 with a fold change more than ± 2, we identified 3,197 differentially expressed genes in TNBCs vs. microdissected normal breast tissue (Supplementary Table 1). To perform drug development studies, we employed network and pathway analysis to identify key targetable pathways using these differentially expressed genes. From this analysis, we observed over-expression of the PI3K/AKT/mTOR (PI3K) pathway whose components included over- expressed AKT, p21, 4E-BP1, S6 Kinase, and others (Fig. 1a, b). Congruent with our RNA-seq data, published analyses from the The Cancer Genome Atlas (TCGA) has also confirmed genomic activation of this pathway even in TNBC samples that do not harbor PIK3CA or PTEN mutations.^22^ To further illustrate this, we used the cBioPortal database (cbioportal.org) to mine the TCGA TNBC data, and as shown in Fig. 1c, the majority of TNBCs harbor a genomic aberration in the canonical components of the PI3K, AKT, and/or PTEN genes.

### Network analysis predicts compensatory Wnt pathway activation

It is well known that single-agent therapy is not always clinically effective, and resistance is common secondary to the activation of compensatory pathways.^23–25^ To this end, we analyzed the RNA-seq data to identify complementary pathways that could be targeted using rational combinations. To do this, we performed an in silico experiment using a new tool called Molecular Activity Predictor (MAP) analysis [a part of the Ingenuity Pathway Analysis (IPA) 9.0 package. The MAP tool enables the prediction of upstream and/or downstream effects of activation or inhibition of molecules in a network or pathway given a starting set of neighboring molecules with “known” activity or expression. This tool leverages the vast literature library present in IPA. As seen in Supplementary Fig. 1, when buparlisib is added in silico (*red color*), it results in certain components of the PI3K pathway to become inhibited (*blue color*) including: PIK3CA, PIK3CB, PIK3CD, PIK3CG and others. The in silico addition of buparlisib (BKM120) also causes certain components of the Wnt pathway to become activated (orange color) including: Wnt ligands (WNT5, WNT6, WNT7, etc), frizzled receptors (FZD), beta-catenin (CTNNB1), LRP1/5/6, and others.

**Fig. 2:**
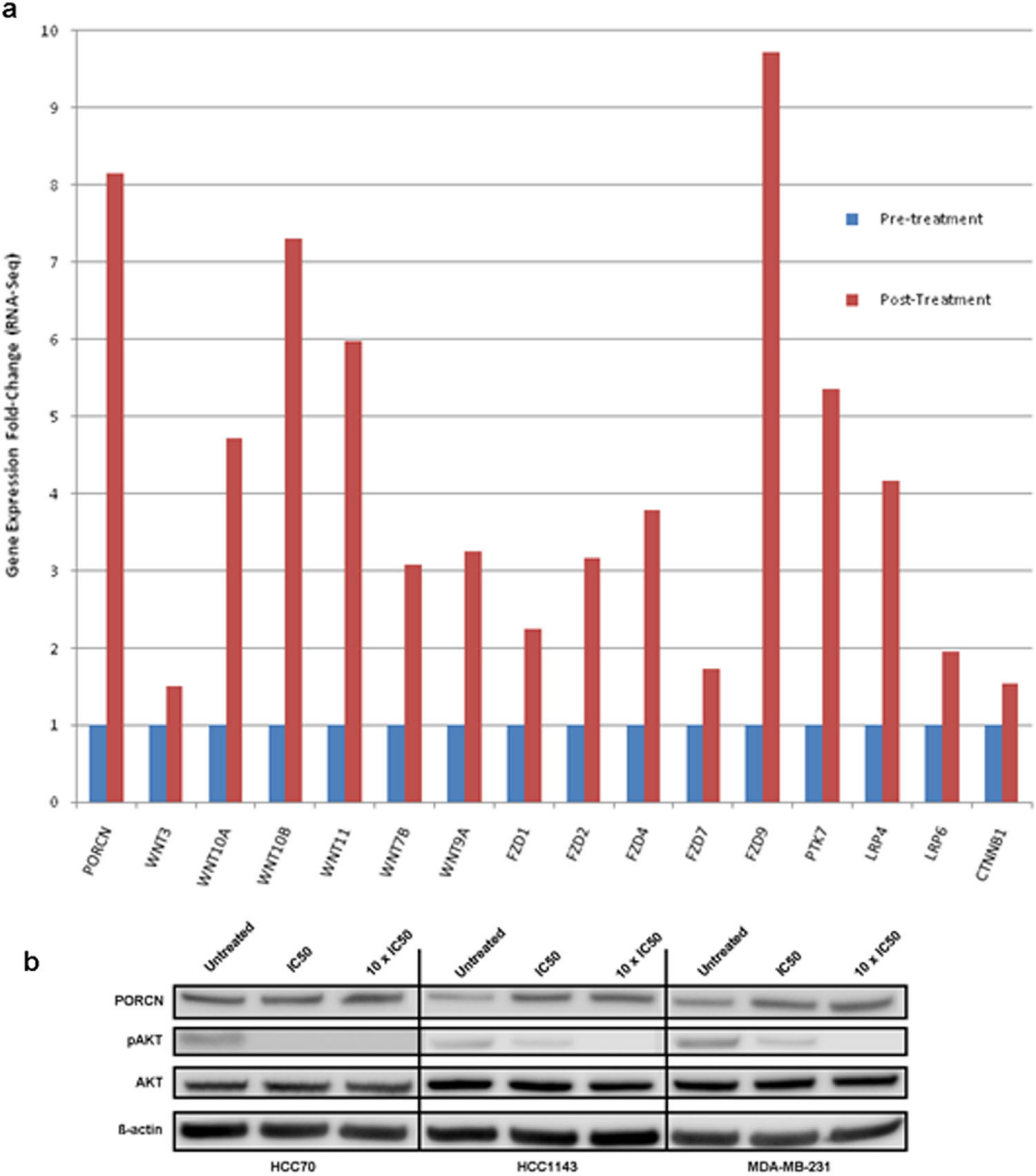
**a** RNA expression of Wnt pathway molecules after buparlisib treatment of the TNBC cell line MDA-MB-231. Cells were treated with buparlisib and RNA-seq (Ampliseq Transcriptome, see methods) was performed on extracted RNA before and after treatment. Genes in the WNT pathway that were observed to have a change in expression include the protein target of WNT974, porcupine (PORCN), as well as the Wnt ligands, FZD receptors, PTK7, LRP4/6, and beta-catenin (CTNNB1). **b** Western blot of porcupine (PORCN), phosphorylated-AKT (p-AKT Ser 473), total AKT, and beta-actin (loading control), before and after treatment of three TNBC cell lines with buparlisib. The western blot shows significant induction of PORCN protein after treatment with buparlisib across all three cell lines

To experimentally confirm the in silico observation of Wnt pathway induction after PI3K inhibition, we produced preliminary data treating the TNBC cell line MDA-MB-231 with buparlisib, and performing RNA-seq before and after treatment. As shown in Fig. 2a, treatment of MDA-MB-231 cells with buparlisib results in a significant increase in the expression of Wnt pathway genes. In Fig. 2a, the expression values of a portion of the Wnt pathway genes are shown, including, porcupine (PORCN), the receptors of Wnt ligands (FZD proteins), protein tyrosine kinase (PTK7), lipoprotein receptor-related proteins 4 and 6 (LRP4, LRP6), β-catenin (CTNNB1), and several WNT ligands (expression values of the full gene list is in (Supplementary Table 2). Druggable genes of interest, PORCN and PTK7, were validated through quantitative polymerase chain reaction (qPCR, Supplementary Fig. 2). Of particular interest, the gene porcupine (PORCN), is ~8-fold over-expressed after PI3K inhibition with buparlisib. Porcupine, is a critical protein involved in Wnt ligand maturation, whose O-acyltransferase activity is required for Wnt ligand pamitoylation and secretion.^21^ More importantly, porcupine is the target of one of only three drugs currently in clinical trial that target the Wnt pathway (clinicaltrials.gov). WNT974, is an oral small molecule inhibitor of porcupine, and recently published data has demonstrated potent inhibition of its target and significant inhibition of tumor growth in vivo.^26^

To further confirm the induction of porcupine after buparlisib treatment, but at the protein level, we performed western blotting for porcupine and p-AKT before and after treatment with buparlisib. Across three TNBC cell lines: MDA-MB-231, HCC70, and HCC1143; treatment with buparlisib resulted in potent inhibition of phospho-AKT as expected (Fig. 2b). Conversely, we observed a significant increase in porcupine protein expression after incubation with buparlisib (Fig. 2b). Quantification of HCC70 PORCN protein only saw a significant increase at 10 × IC50 buparlisib treated (*p*-value 0.0084), while HCC1143 and MD-MB-231 saw significant increases at their corresponding IC50s (*p*-value of 0.013 and 0.0072, respectively) (Supplementary Table 3).

### Dual pathway inhibition with buparlisib + WNT974 demonstrates in vitro synergism

**Fig. 3:**
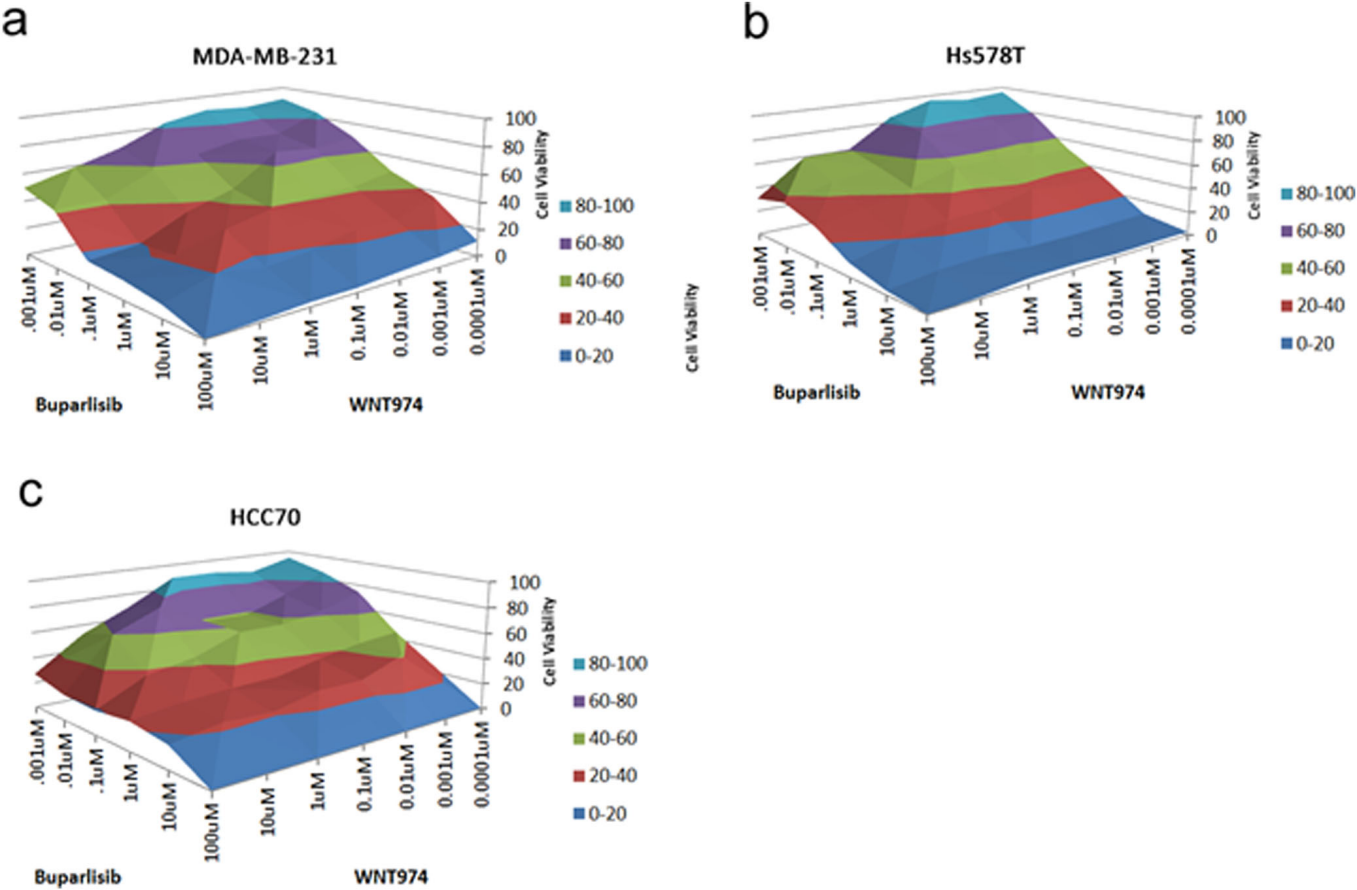
In vitro efficacy of buparlisib and WNT974 across three TNBC cell lines. Buparlisib and WNT974 were dosed at increasing concentrations (*x* and *y*-axes), and the percentage of cell viability was measured (*z*-axis) for cell lines: MDA-MB-231 (**a**), Hs578T (**b**) and HCC70 (**c**). Data is displayed as a cascade plot. Using the Chou–Talalay method, we observed for MDA-MB-231 and Hs578T, a ~50% reduction in cell viability at 100 nM concentration of each drug that was highly synergistic (combination index = 0.33 and 0.36, respectively). For HCC70, we observed an additive effect that was more resistant to the combination with an IC50 of 1 μM

We then went on to test the effect of the combination in reducing TNBC cell viability in vitro. We tested the combination of buparlisib and WNT974 by treating TNBC cell lines MDA-MB-231, Hs578T, and HCC70, with increasing concentrations of each drug. As seen in the cascade plots, all three cell lines were observed to have a significant reduction in cell viability when the treatment was used in combination (Fig. 3a–c). Using the Chou–Talalay method, we observed for MDA-MB-231 and Hs578T, a ~50% reduction in cell viability at 100 nM concentration of each drug that was highly synergistic (combination index = 0.33 and 0.36, respectively).^27^ For HCC70, we observed an additive effect that was more resistant to the combination with an IC50 of 1 μM.

### In vivo pharmacokinetics and pharmacodynamics (PD) of buparlisib + WNT974

In the next step of further developing this combination, we performed an in vivo pharmacokinetic (PK) experiment to assess any potential drug–drug interactions. For PK, 45 NOD-scid IL2Rgamma-null (NSG) mice were given a single dose of buparlisib (*n* = 15), WNT974 (*n* = 15), or the combination (*n* = 15) at pre-established concentrations of 30 mg/kg and 3 mg/kg, respectively.^26, 28^ Plasma was drawn from three mice from each group at 0.5, 1, 2, 6, and 24 h post-dosing. Buparlisib and WNT974 were quantified in the plasma using high-performance liquid chromatography–mass spectrometry (HPLC-MS/MS). PK parameters were largely similar when comparing each drug alone or in combination as demonstrated by similar *C*_max_ concentrations, area under the curves (AUCs), clearance rates (Cl/F) and volumes of distribution (Vdss/F), suggesting the lack of a drug–drug interaction (Supplementary Table 4).

**Fig. 4:**
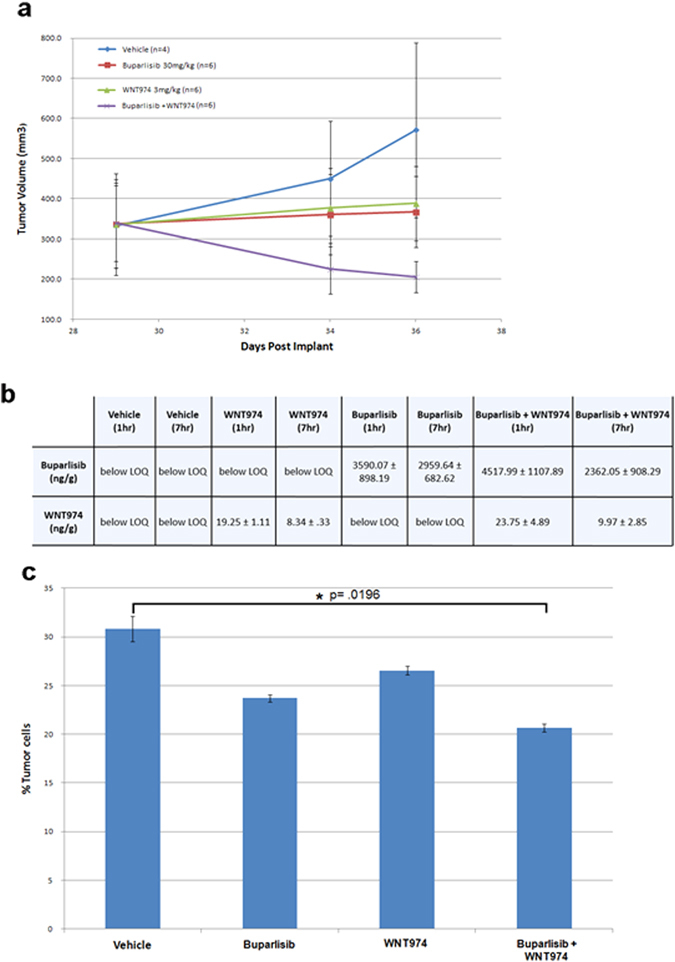
Pharmacodynamic study of buparlisib + WNT974 using cell line xenografts of the TNBC cell line, TMD231 (a MDA-MB-231 cell line variant that metastasizes to the lung). **a** Graph of tumor volumes of NSG mice implanted with TMD-231 cells and treated with vehicle, buparlisib, WNT974, or the combination. After 7 days of treatment, we observe a ~40% decrease in tumor volume. **b** Quantification of drug in tumors resected from mice treated in our PD study. Half of the tumors were resected at 1 h post the last dose, and the other half 7 h post the last dose. Using Highperformance liquid chromatography-mass spectrometry (HPLC-MS/MS), buparlisib and WNT974 were quantified as nanograms of drug per gram of tumor tissue (ng/g). Average values with standard deviation are presented. *LOQ* limit of quantitation **c** As TMD-231 cells have a known propensity to metastasize to the lung, we assessed the effect of the combination therapy on the growth of lung metastases. When compared with the control group, buparlisib and WNT974 as single agents displayed small but non-significant decreases in lung metastases. The combination treatment, however, resulted in a significant decrease in lung metastatic burden compared to the control group (*p* = 0.0196)

To further assess preliminary efficacy and target modulation, a PD experiment was performed. For our PD study, we implanted TMD-231 TNBC cells (a variant of the MDA-MB-231 cell line that metastasizes to the lung) into the mammary fat pad of 24 NSG mice (six mice per group: vehicle, buparlisib, LGK974, buparlisib + WNT974). Tumors were allowed to grow for 29 days to an average size of ~350 mm^3^. Mice were then dosed once per day with buparlisib (30 mg/kg), WNT974 (3 mg/kg), or the combination for 7 days, with intermittent tumor measurements. After only 7 days of treatment, we observed a ~40% decrease in tumor volume with the combination, while tumors actually grew slightly in the face of single agent therapy, again, giving further observations of synergy (Fig. 4a). On the 7th day, the mice had plasma drawn, and then euthanized and the tumor removed. Measurement of buparlisib and WNT974 concentrations in tumor tissue confirmed that these agents were reaching the tumor (Fig. 4b). To assess molecular target modulation, RNA and protein was isolated from each of the tumors in our PD study and biomarker analysis was performed. Inhibition of phospho-AKT, a marker for the activity of buparlisib, was observed in the buparlisib and buparlisib + WNT974 samples, but not in the vehicle or the WNT974 alone samples. (Supplementary Fig. 3a). Similarly, decreased AXIN2 RNA levels (a marker of WNT974 activity) was observed in the WNT974 and combination treated tumors, but not the others (Supplementary Fig. 3b).

The TMD-231 cells used in our PD study have a known propensity to metastasize to the lung.^29^ To assess the effect of the combination therapy on the growth of lung metastases, lung tissues from our PD study were stained and analyzed for metastatic burden. When compared with the control group, buparlisib and WNT974 as single agents displayed small but non-significant decreases in lung metastases (Fig. 4c). The combination treatment, however, resulted in a significant decrease in lung metastatic burden compared to the control group (*p* = 0.0196).

### Buparlisib + WNT974 demonstrates superior survival in TNBC PDX bearing mice

**Fig. 5:**
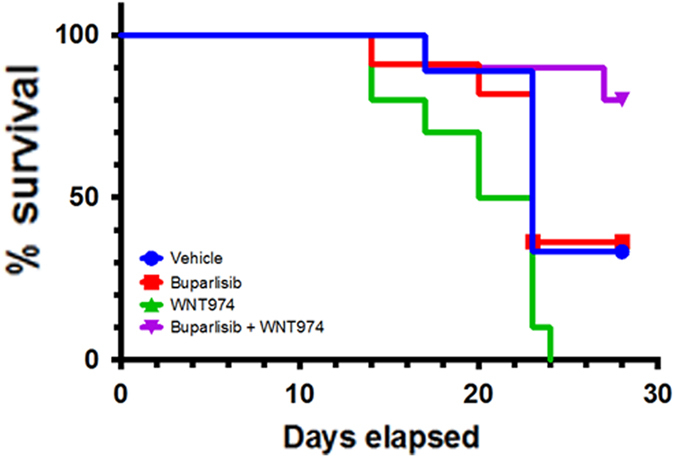
NSG mice were implanted with a TNBC PDX and were treated in four groups (*n* = 10 each): vehicle, buparlisib, WNT974, and the combination of buparlisib + WNT974. At the end of the experiment, 80% of mice treated with the combination survived to the end of the study. In comparison, only 40, 30, and 0% of mice survived treated with buparlisib, vehicle, and WNT974, respectively

In a final efficacy experiment, we tested the buparlisib + WNT974 combination in vivo against a TNBC patient-derived xenograft (PDX). The PDX was selected from Jackson Laboratories based upon its molecular profile (http://tumor.informatics.jax.org/mtbwi/pdxDetails.do?gene=null&variant=null&modelID=TM00091). This tumor was BRCA1 mutated, and had an observed increase in expression in AKT3 and PIK3CA, and a significant decrease in expression of PTEN, demonstrating an active PI3K/AKT pathway. Forty mice were treated in four groups (*n* = 10 each): vehicle, buparlisib, WNT974, and the combination of buparlisib + WNT974. PDX tumors were first grown to an average tumor size of 70–150 mm^3^, and then treated over the course of 28 days. At the end of the experiment, 80% of mice treated with the combination survived to the end of the study (Fig. 5). In comparison, only 40, 30, and 0% of mice survived treated with buparlisib, Vehicle, and WNT974, respectively. Buparlisib is known to have toxicity in humans (depression, anxiety, pneumonitis, and liver toxicity) that may be exacerbated with the addition of WNT974 (whose most common side effect is dysgeusia). To assess potential toxicity of the combination, we measured the body weights of the mice throughout the experiment. Body weights remained consistent between the four treatment groups with a slight average increase in body weight during the course of experiment (Supplementary Fig. 4).

## Discussion

Combination targeted therapies hold significant promise for the treatment of metastatic cancer. Common clinical examples include palbociclib + fulvestrant for metastatic estrogen-receptor-positive breast cancer,^30^ and dabrafenib + trametinib^31^ and nivolumab + ipilimumab^32^ for metastatic melanoma. In the present pre-clinical study, we demonstrate that dual inhibition of the PI3K and Wnt pathway is synergistic against TNBC. Furthermore, our data suggests that the Wnt pathway is activated in a compensatory fashion when TNBC cells are challenged with pan- PI3K inhibition. The hyperactivation of the PI3K pathway is a characteristic seen in many cancers.^33^ It is responsible for the regulation of cell survival, cell cycle progression, and cellular growth.^33^ Of interest, the majority of TNBCs have a genomic aberration in the PI3K/AKT pathway, suggesting this pathway as a precision medicine target. But single-agent PI3K inhibition alone may not be enough to illicit meaningful clinical responses. In this study, we employed a unique approach where the first drug was chosen based on genomic profiling (buparlisib; PI3K), and the second drug based on the compensatory pathway (WNT974, porcupine/Wnt-pathway). This approach is akin to a game of “whack-a-mole”, and suggests a paradigm where hitting both pathways (genomically-aberrant + compensatory) can induce drug synergy.

The compensatory WNT pathway observed in our study is well known for its role in tissue development and cancer metastases.^20^ Of interest, both in silico bioinformatic prediction (based on a large literature database) as well as next generation RNA-sequencing of a TNBC cell line treated with buparlisib saw induction of numerous Wnt pathway molecules including the FZDs, WNT ligands, LRP4/6, PTK7, and porcupine (PORCN). Porcupine induction at the protein level was subsequently confirmed in additional TNBC cell lines. Porcupine (the protein target for WNT974), plays a role in the Wnt ligand maturation process and whose O-acyltransferase activity is required for Wnt ligand pamitoylation and secretion.^21^ WNT974 is currently being tested in phase I clinical trials in a variety of tumor types (clinicaltrials.gov). In an effort to support initiation of future clinical trials of the buparlisib + WNT974 combination, we performed an in vivo PK experiment to demonstrate, that at least in mice, we do not observe a drug–drug interaction when buparlisib and WNT974 are co-administered. Further, in our PD experiment, we observed that both drugs reach the tumor at concentrations that are equivalent to when the drugs are administered individually. Lastly, we demonstrate significant efficacy with the combination both in a cell line xenograft and in an aggressive TNBC PDX model. Crosstalk between the PI3K and Wnt pathways has been previously reported. AKT is known to phosphorylate and inhibit GSK3 leading to increased beta-catenin activity.^34^ More recent data has demonstrated that colorectal tumors that have high levels of beta-catenin are more resistant to PI3K/AKT inhibition.^35^ The exact mechanism of porcupine induction after pan-PI3K inhibition is currently unknown, and warrants further investigation.

In conclusion, we demonstrate a synergistic combination of dual PI3K and Wnt pathway inhibition using buparlisib + WNT974 against triple-negative breast cancer. As drugs against both pathways are currently in clinical trials as single agents, further clinical exploration of dual pathway inhibition is well warranted. Wnt pathway induction after PI3K inhibition provides a novel mechanism of compensatory resistance to PI3K inhibitors that may be applicable to other cancers with characteristic PI3K hyper-activation.

## Methods

### RNA sequencing and pathway analysis

Data from RNA-sequencing of 94 TNBCs compared to 20 normal breast tissues microdissected for ductal epithelium, has been previously described.^18^ Statistically significant differentially expressed genes comparing TNBC vs. normal breast tissues (as reported in Radovich et al.)^18^ were imported into IPA for pathway, network, and upstream regulator analyses (Qiagen, Redwood City, CA, USA). RNA-sequencing for the buparlisib treated MDA-MB-231 cell line experiment was performed as follows. To enrich for the non-ribosomal RNA transcriptome, RNA samples were first depleted of ribosomal RNA using the Low Input Ribominus Kit (Life Technologies). ERCC spike-in controls (Pool 1) were introduced for all samples (Life Technologies). RNA libraries were constructed using the Ion Total RNA-Seq Kit for AB Library Builder System (Life Technologies) per manufacturer’s instructions. Libraries were barcoded using the IonXpress RNA-Seq Barcode 1–16 Kit (Life Technologies), and libraries were quantified using the Agilent TapeStation 2200 along with the DNA D1K Kit. Libraries were diluted to a concentration of 11 pM prior to templating and emulsion polymerase chain reaction (PCR) using the Ion Template OT2 200 v2 kit along with Ion OneTouch 2 instrument (Life Technologies). Templates were quantified using the IonSphere Quality Control Kit (Life Technologies). Samples were sequenced on an Ion Proton Next-Generation Sequencer using the Ion Proton PI chip and the Ion PI Sequencing 200 v2 kit (Life Technologies). Samples were sequenced using two RNA-seq libraries/samples per chip to an average of 30–40 million reads per sample. Reads were mapped to the human genome (hg19) using the STAR algorithm.^36^ Aligned BAM files were then imported into Partek Genomics Suite. RPKM values were called in Partek using the NCBI Refseq database as the gene model.

### In vitro drug sensitivity in TNBC cell lines

Buparlisib (a pan-PI3K inhibitor) and WNT974 (a Wnt pathway inhibitor formerly known as LGK974) were obtained under a Material Transfer Agreement with Novartis (Basel, Switzerland). We assessed PI3K and Wnt pathway drug sensitivity, and synergy on three TNBC cell lines; Hs578T, HCC70, and MDA-MB-231. Cells were maintained either in Dulbecco's modified eagle medium or Roswell Park Memorial Institute medium with 10% FBS, in humidified 5% CO2 incubators. 10,000 cells per well were seeded in 96-well plates and were dosed with increasing log concentrations (1 nM–100 μM) of buparlisib and WNT974 as single-agents and in combination. Cells were treated in a 6 × 6 matrix such that each combination of each dose was tested, with a cascade plot generated from the data. Cell viability was assayed using the Celltiter-Fluor kit (Promega, Madison, Wisconsin) with fluorescence measured using a Synergy 4 microplate reader (BioTek, Winooski, Vermont). The Chou–Talalay method was used to calculate a Combination Index (CI) score to determine synergism.^37^

### In vivo pharmacokinetics and PD

For PKstudies, NSG mice were obtained from the In Vivo Therapeutics (IVT) Core at the Indiana University School of Medicine and given a single dose of buparlisib, WNT974, or the combination at pre-established concentrations of 30 mg/kg and 3 mg/kg respectively.^26, 28^ This experiment employed 45 NSG mice (*n* = 15 for each group). Plasma was drawn from three mice from each group at 0.5, 1, 2, 6, and 24 h post dosage. Buparlisib and WNT974 were quantified using HPLC-MS/MS. PK parameters for buparlisib and WNT974 including AUC, area under the moment curve (AUMC), and half-life (t½) were estimated using noncompartmental methods with Excel^®^. The maximum plasma concentration (*C*_max_) and time of *C*_max_ (*t*_max_) were obtained from the data. The AUC from zero to infinity (AUC_0–∞_) was estimated from the AUC_0–*t*_ (time zero to the last quantifiable concentration *C*_last_) and the AUC from *C*_last_ to infinity, *C*_last_/*k*_el_, where k_el_ is the rate constant of elimination. The AUMC_0–∞_ was estimated by an analogous manner. The clearance (Cl/F, where *F* = bioavailability) of buparlisib and WNT974 were calculated from the dose and AUC_0–∞_. The apparent volume of distribution (Vd_ss_/F) was estimated by the following equation: (dosage/AUC_0–∞_) × (AUMC_0–∞_/AUC_0–∞_). The single-agent PKparameters were compared to the drug combination parameters to determine whether a drug–drug interaction exists. For our pharmacodynamic studies, NSG mice were implanted with TMD-231 TNBC cells into the mammary fat pad of 24 NSG mice (six mice per group: vehicle, buparlisib, WNT974, buparlisib + WNT974). The TMD-231 cell line is a variant of the MDA-MB-231 cell line that metastasizes to the lung. Tumors were allowed to grow to an average size of approximately 350 mm^3^ prior to dosing. To validate compound-mediated modulation of the PI3K/mTOR and Wnt signaling pathways, mice were dosed once a day with vehicle, buparlisib (30 mg/kg), WNT974 (3 mg/kg), or the combination for 7 days. On the 7th day, the mice had plasma drawn, were euthanized, and the tumors were removed. To capture early and late PD effects, half the mice in each group were euthanized with tumors removed at 1 h after the last dose and the other half at 7 h. The drugs in the plasma and tumor tissue were quantified as described previously. All murine studies were approved by the Indiana University Institutional Animal Care and Use Committee (IACUC).

### Western blotting and qPCR validation

Target modulation was assessed using qPCR for Axin2 (a marker of WNT974), and western blot for porcupine and phospho-Akt Ser 473 (a marker of buparlisib activity). qPCR was performed using Taqman assays (Life Technologies). Protein was isolated after 48 h of treatment in biological triplicates using radioimmunoprecipitation assay buffer and quantified with bicinchoninic acid assay (Thermo Fisher). Protein was run on bis-tris gels (Life Technologies) and transferred to polyvinylidene fluoride membrane. Primary pAKT-Ser 473 antibody (Cell Signaling Technology, Beverly, Massachusetts), β-actin (Cell Signaling Technology), and PORCN (Abcam, Cambridge, England) was incubated overnight with subsequent 1-hour incubation with horseradish peroxidase-conjugate secondary antibody. Staining was performed using a chemiluminescent substrate (ThermoFisher). Imaging was performed using an LAS-4000 Luminescent Image Analyzer (Fujifilm, Tokyo, Japan). Quantification of blots was performed using ImageJ (NIH) and statistical analysis using GraphPad.

### Quantification of lung metastases

Lungs were extracted, fixed in 10% formalin, and processed in paraffin. Five-micrometer sections were H&E stained, deparaffinized, blocked, and stained using primary anti-human Ki67 antibody (DAKO, Carpinteria, California). The Aperio whole slide digital imaging system was used for imaging (Leica, Wetzlar, Germany). Using the Aperio ScanScope CS, ×20 images were taken at scan times ranging from 1.5 to 2.25 min. The total nuclear labeling index (Ki67) was generated using the Aperio ImageScope standard positive pixel algorithm. The Image Analysis software was used to calculate the percent of positive pixels (brown staining) in one large cross section from each lung.

### TNBC PDX

NSG mice were trochar implanted in the right hind flank with a passage 1–4, 5 mm^3^ TNBC PDX (ID: BR0901) at The Jackson Laboratory (The Jackson Laboratory, Bar Harbor, Maine). Forty TNBC PDX implanted mice were used (ten mice per group: vehicle, buparlisib (30 mg/kg), WNT974 (3 mg/kg), and buparlisib + WNT974). Tumors were grown to an average of 70–150 mm^3^ and mice averaged 22.5 g body weight. Mice were dosed once-a-day for a maximum of 28 days. Tumor measurements were made two times a week. Mice whose tumors reached a size of 1500 mm^3^ were euthanized per IACUC protocol. To assess toxicity, body weights were measured at the time of tumor measurements.

## Electronic supplementary material

Supplementary Figure 1

Supplementary Figure 2

Supplementary Figure 3

Supplementary Figure 4

Supplementary Table 1

Supplementary Table 2

Supplementary Table 3

Supplementary Table 4
