# Supplementary figures and images for: Dual PI3K and Wnt pathway inhibition is a synergistic combination against triple negative breast cancer
Source: NPJ Breast Cancer. 2017 Apr 26;3:17. doi: 10.1038/s41523-017-0016-8 (PMC5460220; doi:10.1038/s41523-017-0016-8)

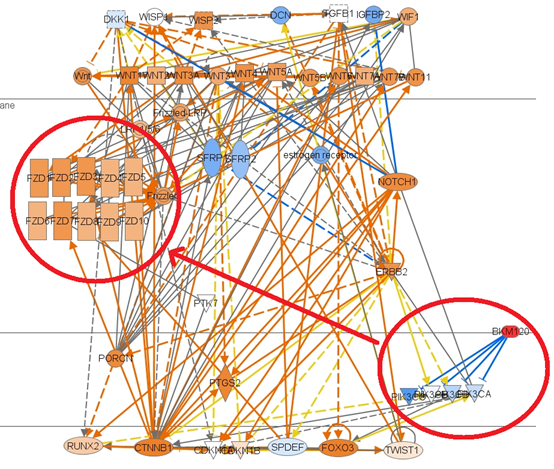

Supplement: Supplementary file 1 — Supplementary Figure 1 [file 41523_2017_16_MOESM1_ESM.tif]

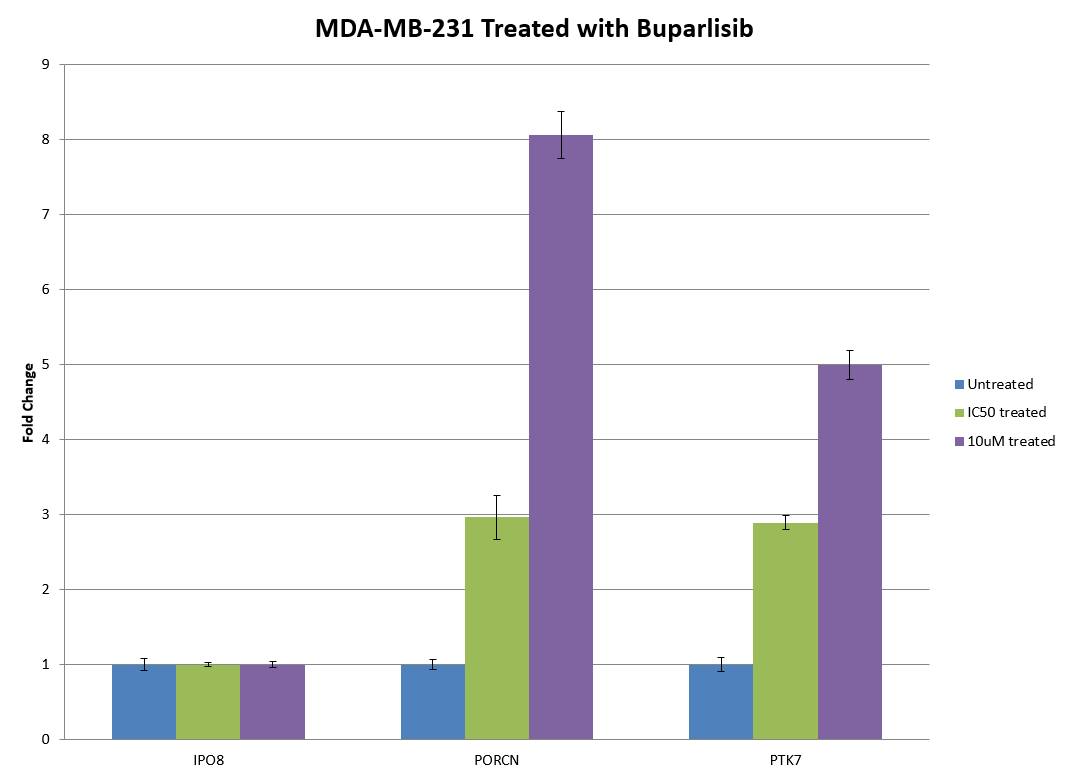

Supplement: Supplementary file 2 — Supplementary Figure 2 [file 41523_2017_16_MOESM2_ESM.tif]

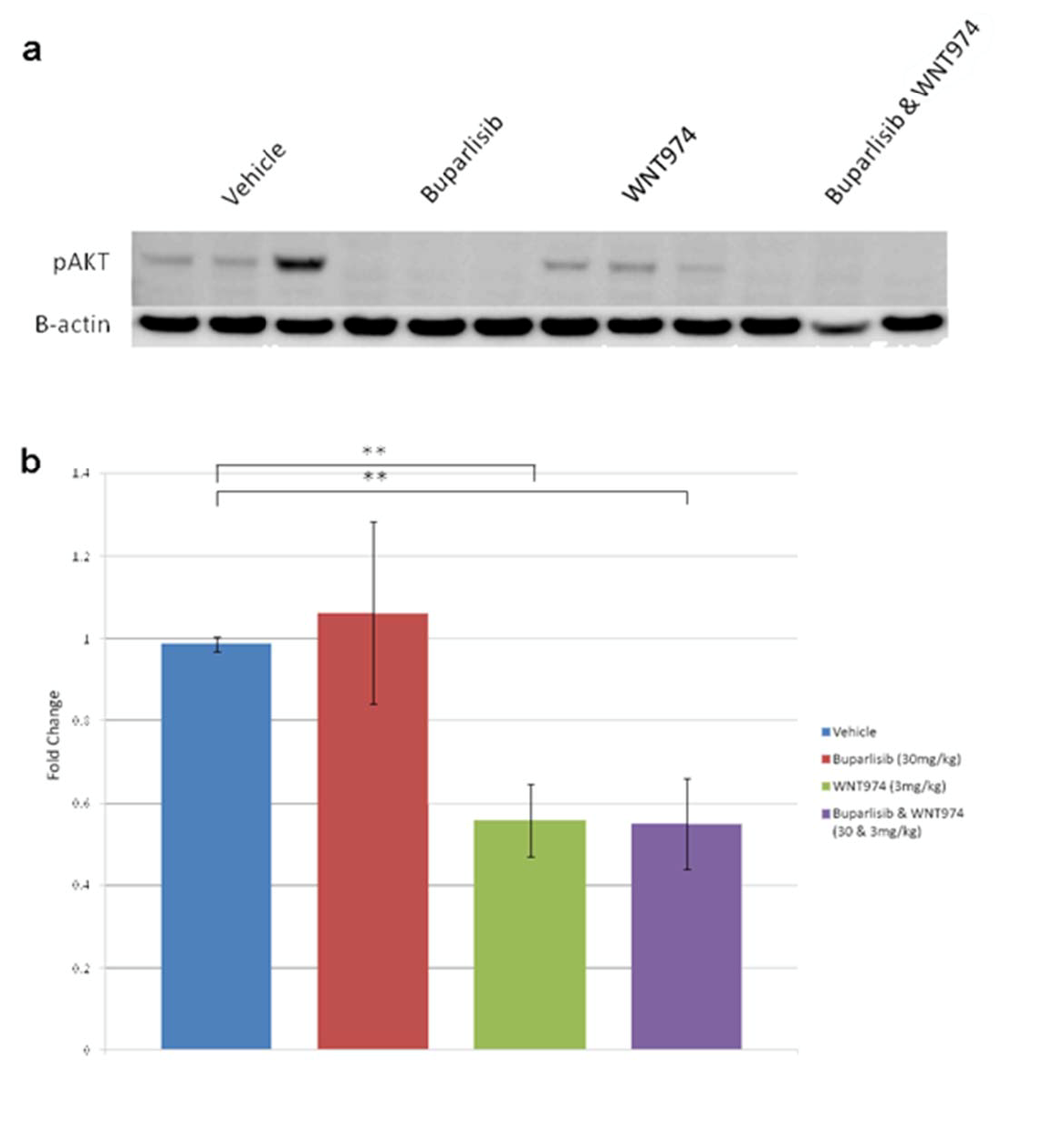

Supplement: Supplementary file 3 — Supplementary Figure 3 [file 41523_2017_16_MOESM3_ESM.tif]

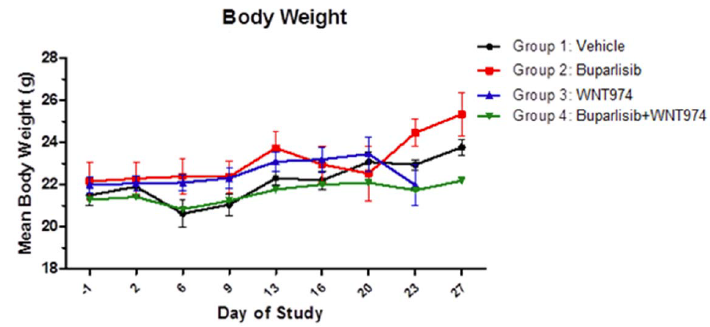

Supplement: Supplementary file 4 — Supplementary Figure 4 [file 41523_2017_16_MOESM4_ESM.tif]

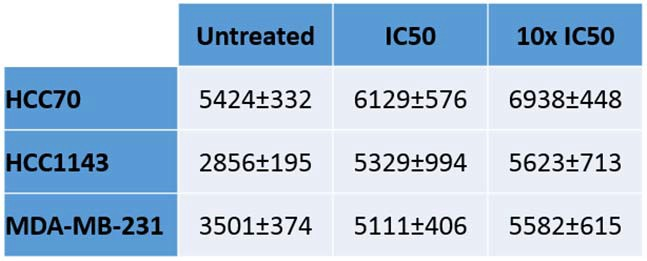

Supplement: Supplementary file 7 — Supplementary Table 3 [file 41523_2017_16_MOESM7_ESM.tif]

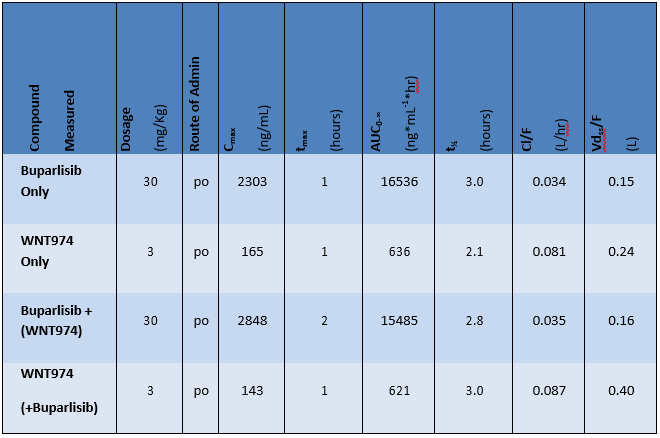

Supplement: Supplementary file 8 — Supplementary Table 4 [file 41523_2017_16_MOESM8_ESM.tif]
